# Effects of vineyard inter-row management on the diversity and abundance of plants and surface-dwelling invertebrates in Central Romania

**DOI:** 10.1007/s10841-019-00215-0

**Published:** 2020-01-14

**Authors:** Cristina Fiera, Werner Ulrich, Daniela Popescu, Claudiu-Ioan Bunea, Minodora Manu, Ioana Nae, Melania Stan, Bálint Markó, István Urák, Andrei Giurginca, Nicole Penke, Silvia Winter, Sophie Kratschmer, Jacob Buchholz, Pascal Querner, Johann G. Zaller

**Affiliations:** 1 Institute of Biology Bucharest, Romanian Academy, 296 Splaiul Independenţei, P.O. Box 56-53, 060031 Bucharest, Romania; 2 Department of Ecology and Biogeography, Nicolaus Copernicus University Toruń, Lwowska 1, 87100 Toruń, Poland; 3 Jidvei Winery, 45 Gării Street, 517385 Jidvei, Alba County Romania; 4 Faculty of Horticulture, University of Agricultural Sciences and Veterinary Medicine, Calea Mănăștur 3-5, 400372 Cluj-Napoca, Romania; 5 Institute of Speleology, Emil Racoviţă” Romanian Academy, 13 Septembrie Street, 050711 Bucharest, Romania; 6 “Grigore Antipa” National Museum of Natural History Şos, Kiseleff 1, 011341 Bucharest 2, Romania; 7 Hungarian Department of Biology and Ecology, Babeș-Bolyai University, Clinicilor 5-7, 400006 Cluj-Napoca, Romania; 8 Department of Environmental Science, Sapientia Hungarian University of Transylvania, Calea Turzii 4, 400193 Cluj-Napoca, Romania; 9 Institute for Integrative Nature Conservation Research, University of Natural Resources and Life Sciences Vienna (BOKU), Gregor Mendel Straße 33, 1180 Vienna, Austria; 10 Institute of Plant Protection, University of Natural Resources and Life Sciences Vienna (BOKU), Gregor Mendel Straße 33, 1180 Vienna, Austria; 11 Institute of Zoology, University of Natural Resources and Life Sciences, Vienna (BOKU), Gregor Mendel Straße 33, 1180 Vienna, Austria

**Keywords:** Vineyard biodiversity, Vascular plants, Viticulture, Trophic groups, Tillage, Arthropods

## Abstract

Vineyard inter-rows are important biodiversity hotspots within agricultural landscapes, especially when they are covered with vegetation. However, little is known on the effects to management intensity on a broad range of surface-dwelling invertebrates and their interaction with vegetation. We assessed the diversity and activity density of ants, beetles, millipedes, mites, spiders, springtails and woodlice using pitfall traps in vineyards with either high management intensity (HI) consisting of frequently tilled inter-rows or low management intensity (LO) with alternating tillage in every second inter-row. The study was performed in the Târnave wine region in Central Romania. We wanted to know whether, (i) vineyard management intensity affects the diversity of plants and invertebrates, and (ii) local habitat characteristics affect species richness of different functional guilds and taxa. Species richness of some invertebrate taxa (Coleoptera, Araneae, Formicidae) did significantly differ between HI and LO vineyards. Only phytophages (some Coleoptera) increased in species richness and activity density with vegetation cover. Vineyard soil properties (organic matter content, pH, P, and K) did not significantly differ between HI and LO vineyards. We conclude that vineyard inter-row management can affect both the conservation of biodiversity and the provision of biodiversity-driven ecosystem services.

**Electronic supplementary material:**

The online version of this article (10.1007/s10841-019-00215-0) contains supplementary material, which is available to authorized users.

## Introduction

Grapevine (*Vitis vinifera* L.) is among the oldest perennial crops and well adapted to (summer-) dry climates. Vineyard inter-rows provide habitats for a range of plant and above- and belowground animal species, especially when covered with vegetation (Kehinde and Samways 2014; Kratschmer et al. 2018). Organisms colonizing these inter-rows provide various ecosystem services (e.g., primary production, pest control, pollination, erosion mitigation and soil nutrient cycling), while their occurrence and abundance are influenced by a range of factors, including tillage practices (Faber et al. 2017), weeds and cover crops, surrounding landscape structures and applications of agrochemicals for pest management (Sharley et al. 2008; Thomson and Hoffmann 2007).

Inter-row management practices are used for weed control and water conservation and can include intensive tillage (resulting in bare soil), alternating tillage, where only every second inter-row is tilled or no tillage with permanent green cover. Instead of or in addition to tillage, the application of herbicides is another common practice to control the inter-row vegetation (Bauer et al. 2004). The respective plant communities determine the physical structure, climatic conditions, food and nesting resources in most habitats and therefore exert considerable influence on the distribution and interactions of animal species (Lawton 1983; McCoy and Bell 1991) and ecosystem services (Winter et al. 2018). Spontaneous vegetation and/or seeded cover crops have a great impact on abundance and diversity of beneficial arthropods in vineyards (Buchholz et al. 2017; Franin et al. 2016). Flowering plants provide nectar and pollen resources to flower-visiting insects (Ambrosino et al. 2006; Kratschmer et al. 2019). Vegetation cover plays an important role in enhancing the abundance and diversity of arthropod predators and consequently reduces pest densities (Rusch et al. 2017). Management of vineyards may affect local biodiversity of plants and invertebrates (Balog and Markó 2007; Caprio et al. 2015; Hadjicharalampous et al. 2002; Simoni and Castagnoli 2007). Vineyard inter-row management has been shown to affect the number and activity of spiders and ground beetles (Norris and Kogan 2000) and alter interactions between collembolans and spiders (Pfingstmann et al. 2019).

In an agricultural landscape including arable crops, vineyards can be important for erosion control, improvement of soil structure and organic matter content and providing suitable habitat for beneficial arthropods (Altieri 2012; Bommarco et al. 2013). Carabid beetles (Coleoptera: Carabidae) are important components of vineyards (Caprio et al. 2015) and they are potentially important natural agents of pest control as they are polyphagous predators, thus they maintain ecosystem functions and services and promote the sustainability of the agroecosystem (Kromp 1999). Spiders (Arachnida: Araneae) also play an important role in biological control of vineyard pests (Bolduc et al. 2005; Bruggisser et al. 2010; Caprio et al. 2015; Costello and Daane 1998; Gaigher and Samways 2014; Isaia et al. 2006). In vineyards spiders can contribute to control of some economically important pests such as grape berry moths (Addante et al. 2003), mealybugs, leafhoppers and planthoppers (Daane et al. 2008). Springtails and mites are also inhabitants of vineyards (Favretto et al. 1992; Nash et al. 2010) and have a wide range of feeding strategies that contribute to soil organic matter decomposition and thus influence the amount of living and dead organic material and nutrient transfers. In vineyards, ants are used as indicators of ecosystem functioning (Chong et al. 2010; De Bruyn 1999). However, some species of ants, spiders and millipedes are also vectors of trunk disease pathogens such as Petri disease and esca which result in reduced grape yield and quality and ultimately significant financial losses (Moyo et al. 2014). Finally, as important decomposers woodlice are also known to be largely affected by local management and associated habitat characteristics, such as soil humidity, pesticide application or tillage operations (Dauber et al. 2005; Paoletti and Hassall 1999).

Ecological studies in vineyards are of particular interest because vineyards are not only agronomically important but also for the conservation of biodiversity (Márquez-García et al. 2019). Vineyards may host many rare and endangered species and biodiversity in general can be high (Costello and Daane 1998; Isaia et al. 2006). For example, the spider species *Erigonoplus globipes* (L. Koch, 1872), which is generally rare in Central Europe (Hänggi et al. 1995), can be commonly found in Romanian vineyards. Therefore, vineyards have a special conservation value and an expanded knowledge on the effects of different management practices and the protection of such habitats is of great importance (Winter et al. 2018).

In the present study, we investigated the effect of different inter-row vegetation management practices on biodiversity in central Romanian vineyards. We studied the cover and diversity of plants and diversity and activity densities of surface-dwelling ants, beetles, millipedes, mites, spiders, springtails and woodlice. We compared effects on these taxa in vineyards with high management intensity (HI) with frequently tilled inter-rows and vineyards with low management intensity (LO) with vegetation cover in every second inter-row and herbicide application in the rows. Our aim was to assess whether (i) vineyard management intensity affects the diversity of plants and invertebrates, and (ii) local habitat characteristics affect species richness of different functional guilds and taxa.

## Materials and methods

### Study area

**Fig. 1:**
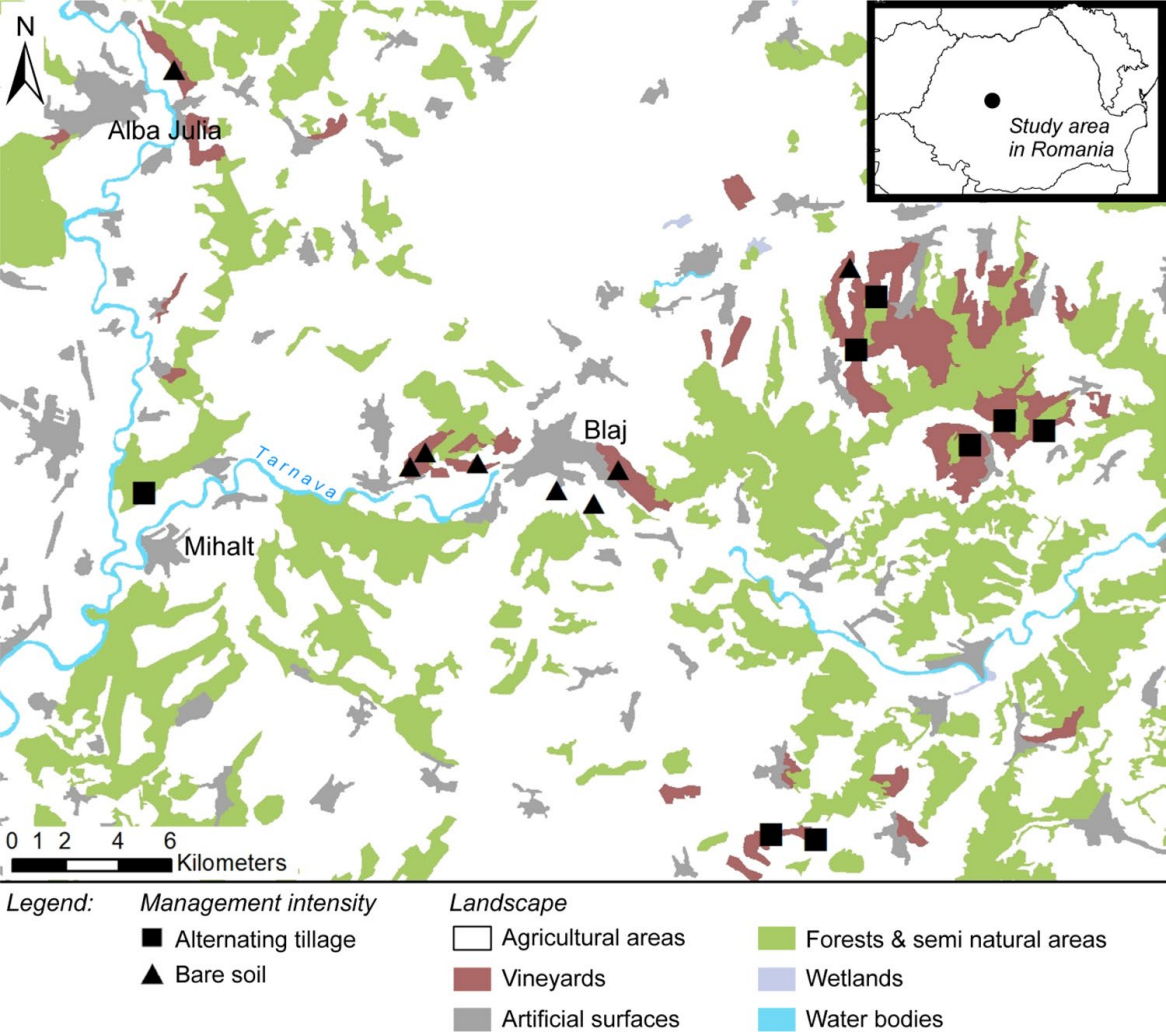
Location of study area in Romanian vineyards. Main map with location of the studied vineyards including respective tillage regime, HI- high and LO-low

The present study was carried out in 16 Romanian vineyards. The study sites were located in the Târnave wine region, a traditional viticulture region in Transylvania, Romania (46.15971° N/23.92991° E; Fig. 1). Most vineyards are under conventional cultivation (> 90%) and are not irrigated. The trellis system consisted of within-row grapevine distances of 1.0 m and inter-row distances varying between 2.15 and 3.00 m. The regional climate is classified as “Dfb” (D: snow, f: fully humid, b: warm summer) after the Köppen–Geiger Climate Classification (Kottek et al. 2006). The dominant soil type is deep brown soil (Marginean et al. 2013). Average annual temperature is 10.8 °C and average annual precipitation is 544.6 mm (https://www.meteoromania.ro/servicii/date-meteorologice/). The vegetation period starts from the beginning of April and lasts until the end of October.

Interviews with wine growers provided information about management practices, such as duration of current management, type of cover crops (spontaneous vs. seed mixture), previous land use type, frequency of herbicide and fungicide application as well as frequency and date of tillage. The raw data used for the present study are given in Table A1 of the electronic supplement S1. Vineyards with high intensive management included different vegetation management practices (tillage frequency inter-row and in-row), fertilization, and weed and fungal control by pesticide application.

### Invertebrate sampling

Invertebrates were sampled between 10 and 22 May 2015. In each vineyard, four pitfall traps (diameter 17 mm, depth 60 mm) were used to collect epigaeic invertebrates (ants, beetles, millipedes, mites, spiders, springtails and woodlice) at each sampling site. Traps were filled with ethylene glycol and a drop of odourless detergent and placed about 6 m distance to each other along a transect in the middle of inter-rows. These traps were left in the field for 12 days. Collected invertebrates were stored in 80% ethanol for further analysis (see Buchholz et al. 2017 for details of the sampling method). Pitfall catches reflect the activity and density of species and results should be presented as activity density (Topping and Sunderland 1992). Almost all sampled specimens were identified to species level (Table B1—of the electronic supplement S2) with the use of taxonomic keys (a list is presented in the electronic supplement S3). Species were assigned to four trophic guilds in accordance with (Gaigher and Samways 2010): phytophages, saprophages, omnivores and predators.

### Soil analysis

We collected soil samples (5.5 cm diameter, 10 cm depth) within inter-row transects in three replicates to determine soil organic matter (SOM) with the Walkley–Black method (SR ISO 14235:2000), soil carbonates with the Scheibler method (SR ISO 10693:1995), and pH potentiometry (SR ISO 10390:2005), phosphorus (P), and potassium (K) with the Egnèr–Riehm–Domingo method Element concentrations were estimated by photo- (STAS 7184/18-80) and calorimetry (STAS 7184/19-82) (Marin et al. 2017) (Table A2—electronic supplement S2).

### Vegetation survey

Vegetation surveys were performed in 2016 in spring (22 April–11 May) and summer (6–15 July) in 12 of the 16 vineyards where invertebrates were sampled 1 year before, because two vineyards were unavailable for sampling and two sites were unexpectedly tilled before vegetation surveys took place. In each vineyard, vegetation surveys were conducted in four 1 × 1 m plots, which were established in the non-tilled inter-rows. Plant species names follow the International Plant Names Index (IPNI). A list of all sampled species can be found in Table B2—electronic supplement S2. Plant trait data for functional diversity indices were obtained from the TRY Database (see Appendix S4 in the online supplementary material for full list of references; Kattge et al. 2011; Kattge et al. 2011) and covered about 66% of species that have been found in the field. For this study, leaf area [mm^2^], dry mass [g/g], specific leaf area [mm^2^], leaf nitrogen content [mg/g], plant height [m] and seed mass [mg] (Table B2—electronic supplement S2) were selected due to their availability and application in other related and recent research projects (Ma and Herzon 2014; Negoita et al. 2016; Ma and Herzon 2014; Negoita et al. 2016; Kazakou et al. 2016; Hall et al. 2020).

### Data analysis

We used ordinary least squares regression for bivariate comparisons of variables, one-way ANOVA to infer differences in diversity, abundances, and plant functional traits between the two management categories, and PERMANOVA (Bray Curtis dissimilarity, Anderson 2001) to assess respective differences in community composition. We used numbers of traps individuals as a proxy for animal abundances. All analyses were performed with Statistica 12.0 (StatSoft, Hamburg, Germany) and Primer 7.0 (PRIMER-e, Auckland, New Zealand).

The indices of functional diversity (richness, evenness, divergence, dispersion (FDis), Rao’s quadratic entropy) were computed in the R environment (R Core Team 2019) using the FD package with plant trait data obtained from the TRY database (Kattge et al. 2011) (Table B2—electronic supplement S2). Functional richness represents the amount of niche space occupied by a community, whereas functional divergence represents how abundance of species is distributed along a trait axis occupied by the community (Mason et al. 2005). β-diversity was calculated from β = γ/α, where γ and α denote total observed richness of a given taxon and the average of local richness, respectively (Whittaker 1972). We excluded millipedes and woodlice species from statistical analysis due to insufficient field data.

## Results

### Characterization of the epigeic invertebrate and plants assemblages

In total, 8728 invertebrate specimens were counted, resulting in 149 operational taxonomic units (OTUs); mainly species (spp.) except for 15 at genus level and some mites at family level: 55 spp. of beetles, 31 spp. of spiders, 24 spp. of springtails, 16 spp. of mites, 15 spp. of ants, 5 spp. of woodlice and 3 spp. of millipedes (Table B1—electronic supplement S2). In total, 2237 individuals from 101 spp. were collected in the LO vineyards and 6491 individuals from 106 spp. in the HI vineyards. Mean activity density of epigeic dwelling invertebrates was 86.13 ± 98.37 individuals per LO site and 31.25 ± 45.84 individuals per HI site. Among beetles, the families Carabidae (10 spp.) and Staphylinidae (9 spp.) were the most diverse families. Collembola were the most abundant (53.7% of the material collected), followed by ants (31.5%).

Phytophages were represented by beetles of the families Tenebrionidae, Anthicidae, Cerambycidae, Cetoniidae, Curculionidae and Elateridae. Formicidae were omnivores. Predators were mainly represented by Arachnida, including Acari (1.2%) and Araneae (31.5%); Coleoptera, with: Carabidae (1.2%), Staphylinidae, Cantharidae, Coccinellidae and Monotomidae less than 1%). Saprophages were comprised mostly of Collembola (53.7%), Oniscoidea (6.3%), Diplopoda (1%) and mites (0.7%) in the families: Achipteriidae, Ceratozetidae, Phenopelopidae, Liacaridae, Scheloribatidae and Tectocepheidae. The invertebrate assemblages were mainly dominated by saprophages (mainly Collembola) and omnivores (ants), with these two trophic groups representing 85.2% of the total animals collected. Predators were the third most abundant group, representing 4.13% of the total catches. They were primarily comprised of generalist predators such as Araneae, Coleoptera (mainly Carabidae and Staphylinidae) and some groups of mites. In Araneae, the dominant family was Lycosidae (wolf spiders). Within the predator trophic group, we also noted the presence of *Erigonoplus jarmilae* (Miller, 1943), which is a new record for the Romanian spider fauna. This species was known before only from Austria, Czech Republic, Slovakia, Albania and Russia (Nentwig et al. 2019). This rare species prefers the ground layer or vertical surfaces of the very dry, warm, open habitats, between 200 and 500 m elevation (Buchar and Růžička 2002). The intensively managed vineyards (HI vineyards) harbored more species of animals and larger populations (except Coleoptera) than the vineyards covered with vegetation (LO vineyards) (Table 1). Relative species overlap was only 59% (Table 1).

**Table 1:** Total species richness (S) and activity densities (D) of animal groups used in statistical analysis, for plants D refers to mean vegetation cover, beta-diversity (β = γ/α), and the absolute and relative species overlap of major plant and animal taxa and of feeding guilds in vineyards of high and low management intensity

Taxon/trophic guild	High		Low		β-diversity		Absolute species overlap	Relative species overlap
	S	D	S	D	High	Low		
Plantae	55	54.6	91	78.2	2.02	3.14	42	0.76
Acari	12	54	11	55	4.80	5.87	3	0.27
Araneae	27	145	12	37	4.70	9.60	8	0.67
Coleoptera	27	103	44	248	3.66	3.01	16	0.59
Collembola	22	3500	20	1192	2.63	2.96	18	0.90
Formicidae	13	2269	9	484	2.66	3.13	7	0.78
Omnivores	13	2269	9	484	2.66	3.13	7	0.78
Phytophages	9	30	14	30	5.14	6.22	4	0.44
Predators	43	211	37	188	4.71	4.23	17	0.46
Saprophages	43	3981	44	1535	3.58	5.10	12	0.72
All animals	104	6491	100	2237	3.63	4.00	59	0.57

A total of 99 taxa of plants were identified to species level, which belong to 27 families and five were identified at genus level (Table B2—electronic supplement S2). Most recorded taxa belonged to the Asteraceae family (17 species) followed by the Poaceae family (13 species) with the highest average relative cover of 42.7 ± 20.5% in LO and 33.4 ± 18.5% in HI managed vineyards. Lamiaceae (10 species) and Scrophulariaceae (8 species) were also frequently found in HI and LO vineyards, whereas Fabaceae (8 species) were more frequent in LO vineyards.

Furthermore, we found significant differences in plant community composition and vegetation cover between HI and LO managed vineyards (Table 2). We did not find significant effects of management intensity on plant species richness, abundance, and important species traits (Table 2), as well as on functional diversity (richness, evenness, divergence, dispersion, Rao’s quadratic entropy, not shown). Community composition of species plants between vineyards varied more than between less intensively managed vineyards when using β-diversity for comparison (Table 1). Only two intensively managed vineyards had significantly less vegetation cover than the LO-vineyards (Table B2—electronic supplement S2).

**Table 2:** One way PERMANOVA and ANOVA to detect differences between vineyards of high and low management intensity for major plant community variables (N = 48). Significant differences are shown in bold type

Variable	Method	Management intensity	
		F	P
Community composition	PERMANOVA	**3.29**	** < 0.001**
Species richness	ANOVA	0.01	0.94
Abundance	ANOVA	0.66	0.42
Specific leaf area	ANOVA	0.28	0.59
Seed mass	ANOVA	0.95	0.33
Vegetation cover	ANOVA	**22.4**	** < 0.001**

Species richness of some invertebrate taxa (Coleoptera, Araneae, Formicidae) did significantly differ between HI and LO vineyards (Table 3). Activity densities of Collembola and Araneae did significantly differ between HI and LO vineyards (Table 3).

**Table 3:** One way PERMANOVA (community composition) and ANOVA (richness and densities) to detect differences in management intensity (high − low) for major animal taxa (N = 12). Significant differences are shown in bold type

Taxon	Community composition		Species richness		Densities	
	F	P	F	P	F	P
Coleoptera	**2.78**	** < 0.01**	**4.52**	**0.05**	4.07	0.06
Araneae	1.46	0.11	**5.34**	**0.04**	**8.85**	**0.01**
Collembola	1.40	0.18	1.09	0.31	**7.75**	**0.01**
Formicidae	0.47	0.91	**6.63**	**0.02**	2.26	0.16
Acari	0.96	0.54	0.51	0.38	0.01	0.97
All animals	**2.26**	** < 0.01**	0.99	0.36	**7.97**	**0.01**

### Local habitat characteristics on epigeic invertebrates

**Fig. 2:**
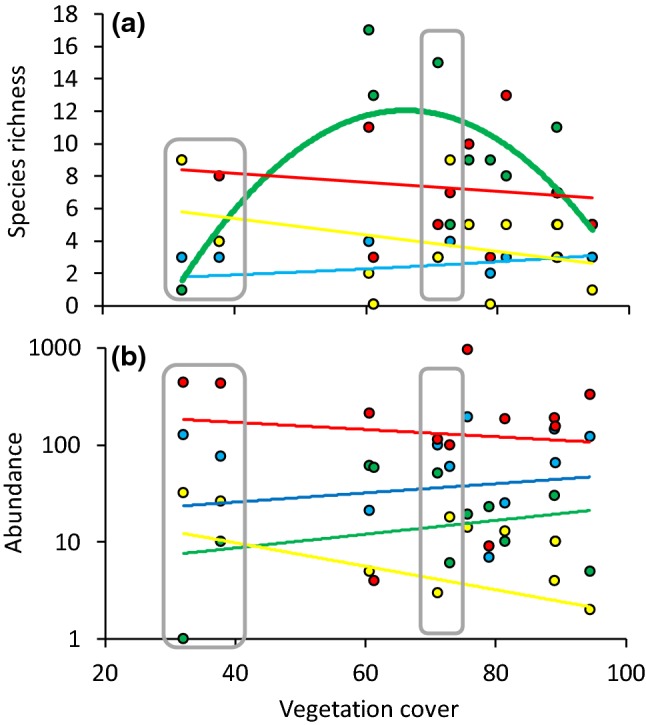
Species richness (**a**) and activity density of (**b**) Coleoptera (green), Formicidae (blue), Araneae (yellow), and Collembola (red) in response to mean vegetation cover. Ordinary least squares linear (**a**) and exponential (**b**) regression lines are not significant at the 5% error level. The bold green line in **a** refers to a second order polynomial regression where the quadratic term is significant at P < 0.05. The grey rectangles mark the four sites of intense management. (Color figure online)

**Fig. 3:**
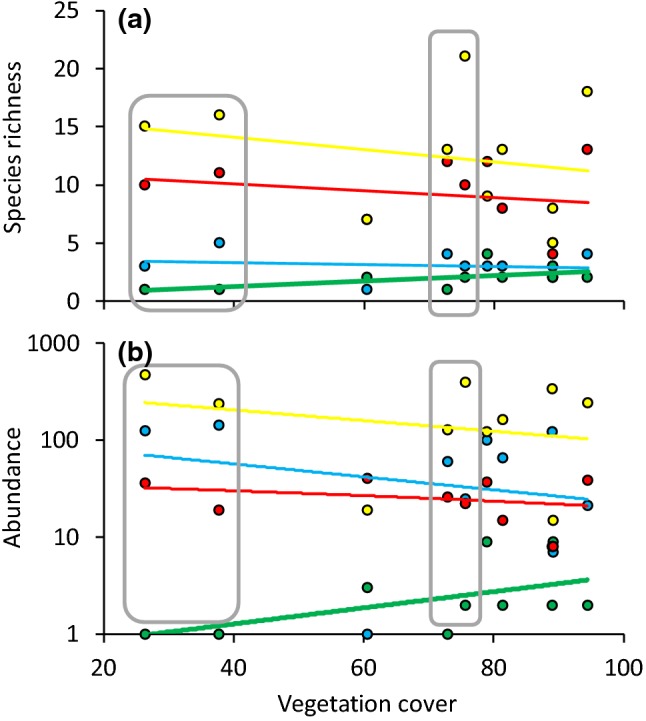
Species richness (**a**) and activity density of (**b**) omnivores (blue), saprophages (yellow), predators (red) and phytophages (green) in dependence on mean annual vegetation cover. Ordinary least squares linear (**a**) and exponential (**b**) regression lines are not significant at the 5% error level. The bold green lines in **a** and **b** refer to significant regressions at P < 0.05. The grey rectangles mark the four sites of intense management. (Color figure online)

We did not find a consistent influence of vegetation cover on invertebrate species richness and activity density (Fig. 2). Coleoptera seemed to prefer intermediate plant cover (Table 2). Except for phytophages that increased in species richness and activity density with plant cover (Fig. 3), species richness and abundance of the other animal trophic groups did not significantly increase with vegetation cover (Table 2).

Vineyard soil properties (organic matter content, pH, P, and K) did not significantly differ between HI and LO vineyards (one-way ANOVA: all P(F_1,13_) > 0.05—Table A2, electronic supplement S1). Vegetation cover did correlate positively with soil organic matter content (r = 0.51, permutation P < 0.05). Except for mites where we found strong positive correlations of species richness (r = 0.85, permutation P < 0.01) and activity density (r = 0.88, permutation P < 0.01) with soil organic matter content, invertebrate species richness and abundances were not significantly linked to soil properties.

## Discussion

### Different effects of vineyard tillage intensity on the diversity of plants and invertebrates

The results of this study show that vineyard management intensity affect the diversity of plants and some invertebrate groups. Lower tillage intensity resulted in higher plant diversity and plant abundance (Table 1), which also increased above-ground biomass in a French study (Kazakou et al. 2016). Vegetation cover differed significantly between vineyards of different management intensity. These findings are relevant for the provision of ecosystem services such as erosion mitigation or carbon sequestration via enhanced higher vegetation cover (Biddoccu et al. 2016; Guzmán et al. 2019; Ruiz-Colmenero et al. 2013). Relative coverage of annual and perennial species also showed a significant response to management intensity, with greater coverage by annuals in vineyards with high management intensity. Tillage destroys the current vegetation cover but it also creates beneficial conditions for seedling establishment of annual species (Gago et al. 2007) such as *Amaranthus powelii* S. Wats., *Chenopodium album* L. agg. and *Portulaca oleracea* L. Perennial plants characteristic of grasslands and therefore less tolerant to tillage such as *Lolium perenne* L., *Plantago lanceolata* L. and *Trifolium repens* L., benefit from mulching in low management intensity vineyards. Consequently, plant community composition of the two inter-row management intensities was significantly different (Table 2). Species diversity of plants and consequently functional diversity clearly increased with less frequent tillage (Tables 1, 2). Functional diversity of plants was also highest in vineyards with lower tillage frequency and spontaneous vegetation in French vineyards (Kazakou et al. 2016).

Despite the similarity of invertebrate assemblages between the two vineyard management systems (HI and LO), we found differences in terms of species diversity and density. Vineyards with high tillage intensity supported higher levels of diversity of invertebrates (with one exception: Coleoptera) than did LO vineyards. Contrary to our study, carabid species (brachypterous group of Coleoptera) were negatively correlated with grass cover and grass height (Caprio et al. 2015). In general, evidence of the effects of management practices on vineyard biodiversity is ambiguous. Several studies described a decline in biological diversity, measured as species richness, the abundance of species or other measures of community structure in high managed vineyards compared to less managed ones: carabid insects and spiders (Caprio et al. 2015). Springtails appeared to be more sensitive to tillage intensity (Buchholz et al. 2017) than to either residue management or N fertilization (Coulibaly et al. 2017). Also, oribatid mites were sensitive to mechanical cultivation of soil (Seniczak et al. 2018). Ant assemblages were negatively affected by soil tillage in vineyards (Sharley et al. 2008).

In some South African vineyards, saprophage communities were most probably favoured by the enhanced detritus-based food webs resulting from increased organic amendments and reduced fungicides (Gaigher and Samways 2010). Although we did not explicitly sample soil arthropods, our findings are in line with others suggesting that soil arthropod biodiversity and functioning is often dependent on agricultural management (Diekötter et al. 2010). Therefore, soil arthropods should be considered as a valuable resource that requires adequate habitat management so that the ecosystem services they supply are enhanced (Gonçalves et al. 2018).

### Local habitat characteristics affect species richness of different functional guilds and taxa

In this study, we found that reduced tillage intensity in the LO vineyards contributed to higher diversity of plants. The roots and litter provided by cover crops can supply the appropriate microhabitats for soil phytophages, thus benefiting their populations, which in turn can promote the increase of predators, also resulting in an increase of soil biological diversity (Komatsuzaki 2008). The review of Puig-Montserrat et al. (2017) highlighted the importance of vegetation diversity for enhancing populations of beneficial arthropods in vineyards. Woodcock et al. (2008) showed the positive effects of composition and diversity of plants around the field margins on ground beetle diversity. Our study also showed that phytophages increased in species richness and activity density with plant cover. Fields with dense vegetation cover and high plant diversity usually have more predaceous and parasitic arthropods than weed-free fields (Speight and Lawton 1976). Indeed, higher arthropod diversity was associated with increased compositional and structural diversity of the vegetation in vineyards where vegetation cover was present (Gaigher and Samways 2010). Vineyards that have ground vegetation cover with high plant species diversity have higher activity densities and richness of phytophages (Gonçalves et al. 2018). A diverse plant community can influence beneficial arthropod populations by providing food or habitat resources that might not be found in a simple plant community (Costello and Daane 1998). An increase in plant species richness can potentially support increasing numbers of specialized consumers (Siemann 1998), which in turn can encourage a greater diversity of predators through cascade effects (Hunter and Price 1992).

In a study in Australian vineyards, carabids were positively affected by ground cover consisting of compost and straw, whereas staphylinids were not affected (Thomson and Hoffmann 2007). Carabids seem to be negatively affected by deep ploughing and enhanced by reduced tillage frequency (Kromp 1999). Spider abundance might be affected by small-scale habitat structure for web building (Alaruikka et al. 2002) as some Linyphiidae utilize sheet webs located on or near the ground (Thornhill 1983). Also, the proportion of non-crop land in an agricultural landscape has been shown to influence the abundance of spiders (Schmidt et al. 2008). Predators in vineyards benefit from reduced management intensity and increased heterogeneity (Isaia et al. 2006; Sharley et al. 2008). Agricultural practices have a greater effect on spiders than other invertebrate groups (Isaia et al. 2006; Jeanneret et al. 2003).

The negative correlation between omnivore richness and the percentage of ground cover suggested that vineyards with a higher percentage of ground cover were expected to have lower omnivore richness (Gonçalves et al. 2018). In other studies, ground cover positively influenced the activity of carabids (Cole et al. 2005; Saska et al. 2014), as did the maintenance of mowing residues at the surface (Shearin et al. 2008). Surface mulches may also influence the abundance of a range of invertebrates. Abundance of ground beetles, parasitoid Hymenoptera and spiders collected with pitfall traps were increased by the addition of mulches (Thomson and Hoffmann 2007). Ground cover (weeds and mowing residue) have been shown to be important in the enhancement of ant populations (White et al. 2011). Tests of different mulch material in vineyards show that springtails and ants were most affected (Addison et al. 2013).

Microarthropods, in particular, mites and springtails, are a major component of soil biota and are known to be important contributors to soil formation, organic matter transformation (mites—this study), nutrient cycling, C accumulation and plant and microbial diversity (Costantini et al. 2015). Besides, organisms which are directly associated with vineyard crops (e.g., mites living on grape leaves) presumably are exposed to a generally higher disturbance level (Peverieri et al. 2009) than the taxa we studied in this study. Different taxa react differently not only to an increase in disturbance, but also to different types of intensities (Abensperg-Traun et al. 1996; Zulka et al. 1997).

## Conclusions

Our results demonstrate that inter-row management practices affect vegetation cover, diversity of plants and some invertebrates. This can increase the provision of ecosystem services in Romanian vineyards, such as the conservation of species, soil erosion mitigation and potentially natural pest control. In agricultural landscapes, vineyards can harbor otherwise rare species (Altieri and Nicholls 2002; Schmitt et al. 2008) as also shown in this study where the spider *Erigonoplus jarmilae* (Miller, 1943) was found as a new record for Romania. An identification of practices that encourage the persistence of rare species and the maintenance of natural enemy abundance may therefore help to reduce the necessity for insecticide applications.

## Electronic supplementary material

Below is the link to the electronic supplementary material.

Supplementary file1 (DOCX 25 kb)

Supplementary file2—Plant and invertebrate communities from Romanian vineyards (HI and LO managed). (XLSX 56 kb)

Supplementary file3 (DOCX 27 kb)

Supplementary file4 (DOCX 25 kb)
