# Supplementary material for: Effects of vineyard inter-row management on the diversity and abundance of plants and surface-dwelling invertebrates in Central Romania
Source: J Insect Conserv. 2020 Jan 14;24(1):175–85. doi: 10.1007/s10841-019-00215-0 (PMC7002328; doi:10.1007/s10841-019-00215-0)
Supplement: Supplementary file 1 — Supplementary file1 (DOCX 25 kb) [file 10841_2019_215_MOESM1_ESM.docx]

**Effects of vineyard inter-row management on the diversity and abundance of plants and surface-dwelling invertebrates in Central Romania**

Cristina Fiera^1^, Werner Ulrich^2^, Daniela Popescu^3,4^, Claudiu-Ioan Bunea^4^, Minodora Manu^1^, Ioana Nae^5^, Melania Stan^6^, Bálint Markó^7^, István Urák^8^, Andrei Giurginca^5^, Nicole Penke^9^, Silvia Winter^9,10^, Sophie Kratschmer^9,11^, Jacob Buchholz^11^, Pascal Querner^11^, Johann G. Zaller^11^*

***S1. Vineyards features from Târnave region, Romania***

**Table A1: Management practices in Romanian vineyards**

| **Landscape**  **ID** | **Vineyard ID** | **Locality** | **Longitude** | **Latitude** | **Management**  **intensity** | **How long are the vineyard cultivated as vineyards?** | **What was before the vineyard cultivation?** | **How long is the current Management**  **applied in the vineyards?** | **Frequency of fungicide^1^** | **Frequency of chemical herbicides^2^ in-row vegetation** | **Mechanic –**  **harrowing,cutting,**  **mulching between**  **row**  **vegetation** |
| --- | --- | --- | --- | --- | --- | --- | --- | --- | --- | --- | --- |
| 1 | HI | Ciumbrud | 46.32064 | 23.76167 | Bare soil | since 1980 | vineyard | from 2011 | 8 | 2 | 2 |
| 2 | LO | Mihalț | 46.1795 | 23.7196 | Permanent vegetation | from 2008 | grassland | from 2010 | 0 | 0 | 2 |
| 3 | HI | Craciunelul de Jos | 46.18076 | 23.85841 | Bare soil | more than 40 years | vineyard | more than 11 years | 7 | 2 | 2 |
| 4 | HI | Craciunelul de Jos | 46.17650 | 23.84996 | Bare soil | from 2006 | fruit tree plantation (apples) | from 2006 | 7 | 2 | 2 |
| 5 | HI | Craciunelul de Jos | 46.17469 | 23.88289 | Bare soil | more than 45 years | vineyard | from 1971 | 7 | 0 | 2 |
| 6 | HI | Blaj | 46.15971 | 23.92991 | Bare soil | from 2012 | other crops (corn, cereals, etc) | from 2012 | 9 | 1 | 3 |
| 7 | HI | Blaj | 46.15579 | 23.93625 | Bare soil | more than 35 years | vineyard | from 1980 | 9 | 1 | 3 |
| 8 | HI | Blaj | 46.16575 | 23.95033 | Bare soil | more than 45 years | vineyard | from 1970 | 7 | 0 | 2 |
| 9 | LO | Cenade | 46.03583 | 24.00887 | Alternating tillage | from 2008 | grassland | from 2012 | 10 | 1 | 3 |
| 10 | LO | Cenade | 46.03225 | 24.02165 | Alternating tillage | from 2008 | grassland | from 2012 | 10 | 1 | 4 |
| 11 | LO | Tăuni | 46.1577 | 24.1225 | Alternating tillage | from 2006 | other crops (corn, cereals, etc) | from 2009 | 9 | 2 | 3 |
| 12 | LO | Tăuni | 46.1643 | 24.1408 | Alternating tillage | from 2007 | grassland | from 2010 | 9 | 2 | 3 |
| 13 | LO | Tăuni | 46.1518 | 24.1524 | Alternating tillage | from 2007 | grassland | from 2010 | 9 | 2 | 5 |
| 14 | LO | Jidvei | 46.1135 | 24.0431 | Alternating tillage | more than 40 years | vineyard | from 2013 | 12 | 1 | 4 |
| 15 | LO | Jidvei | 46.1216 | 24.0438 | Alternating tillage | more than 40 years | vineyard | from 2013 | 10 | 2 | 4 |
| 16 | HI | Jidvei | 46.1323 | 24.0433 | Bare soil | from 2010 | grassland | from 2010 | 11 | 2 | 3 |

1. values refer to the average number of times fungicide treatment was performed in the previous three years with Mancozeb, Cyazofamid, Disodium phoshonate, Folpet, Mandipropamide, Myclobutanil, Tryfloxystrobin, Tebuconazole, Pyrimethanil.
2. values refer to the average number of times chemical herbicides treatment was performed in the previous three years with: Flazasulfuron, Glufosinate, Glyphosate.

**Table A2: Soil properties in Romanian vineyards.**

| **Landscape ID** | **Vineyard ID** |  | **Soil organic matter (%)** | **pH** | **CaCO3** | **Phosphor (ppm)** | **Potassium (K)** |
| --- | --- | --- | --- | --- | --- | --- | --- |
| **1** | HI |  | 2.27 | 7.74 | 5.2 | 30 | 530 |
| 2 | LO |  | 2.69 | 7.85 | 5.8 | 27 | 280 |
| 3 | HI |  | 2.54 | 7.4 | 7.5 | 36 | 154 |
| 4 | HI |  | 2.49 | 7.55 | 7.8 | 27 | 280 |
| 5 | HI |  | 1.41 | 7.83 | 2.9 | 51 | 456 |
| 6 | HI |  | 6.56 | 5.86 | 4.49 | 5 | 128 |
| 7 | HI |  | 1.09 | 7.48 | 0.7 | 41 | 406 |
| 8 | HI |  | 0.61 | 7.94 | 3.1 | 51 | 224 |
| 9 | LO |  | 4.91 | 5.88 | 4.49 | 4 | 88 |
| 10 | LO |  | 5.91 | 5.69 | 4.49 | 5 | 162 |
| 11 | LO |  | 1.76 | 4.9 | 4.49 | 20 | 114 |
| 12 | LO |  | 2.07 | 7.76 | 1 | 18 | 268 |
| 13 | LO |  | 2 | 7.62 | 1.1 | 27 | 168 |
| 14 | LO |  | 1.56 | 6.23 | 4.49 | 51 | 470 |
| 15 | LO |  | 2.2 | 7.77 | 5.6 | 48 | 460 |
| 16 | HI |  | 2.69 | 7.83 | 8.7 | 85 | 620 |
