# Supplementary material for: Effects of vineyard inter-row management on the diversity and abundance of plants and surface-dwelling invertebrates in Central Romania
Source: J Insect Conserv. 2020 Jan 14;24(1):175–85. doi: 10.1007/s10841-019-00215-0 (PMC7002328; doi:10.1007/s10841-019-00215-0)
Supplement: Supplementary file 3 — Supplementary file3 (DOCX 27 kb) [file 10841_2019_215_MOESM3_ESM.docx]

**Effects of vineyard inter-row management on the diversity and abundance of plants and surface-dwelling invertebrates in Central Romania**

Cristina Fiera^1^, Werner Ulrich^2^, Daniela Popescu^3,4^, Claudiu-Ioan Bunea^4^, Minodora Manu^1^, Ioana Nae^5^, Melania Stan^6^, Bálint Markó^7^, István Urák^8^, Andrei Giurginca^5^, Nicole Penke^9^, Silvia Winter^9,10^, Sophie Kratschmer^9,11^, Jacob Buchholz^11^, Pascal Querner^11^, Johann G. Zaller^11^*

***S3. Taxa keys used for identification of invertebrates***

The reference collection of invertebrate specimens is held at Institute of Biology Bucharest, Romanian Academy (Collembola/Acari), “Emil Racoviţă” Institute of Speleology of the Romanian Academy, Bucharest (Oribatid mites, Izopoda, Diplopoda), „Grigore Antipa” National Museum of Natural History, Bucharest (Coleoptera), Hungarian Department of Biology and Ecology, Babeș-Bolyai University, Cluj-Napoca (ants), Sapientia Hungarian University of Transylvania, Cluj-Napoca (spiders).

The following keys were used for identification of invertebrates:

**Coleoptera**

1. Assing V., Schülke M., 2011. Freude-Harde-Lohse-Klausnitzer - Die Käfer Mitteleuropas, Band 4. Staphylinidae I. Zweite neubearbeitete Auflage. Heidelberg:Spektrum Academischer Verlag, I-XII, 1-560.
2. Baraud J., 1988. Présence d’une troisième espèce de *Pleurophorus* Mulsant dans la faune de France (Coleoptera: Aphodiidae). L’Entomologiste, 44 (5): 253-256.
3. Gîdei P., Popescu I.E., 2009. Îndrumător pentru cunoașterea coleopterelor. Editura Pim, Iași, 5-419.
4. Gîdei P., Popescu I.E., 2012, Ghidul coleopterelor din România I. Editura Pim, Iași, 5-533.
5. Hůrka K., 1996, Carabidae of the Czech and Slovak Republics. Illustrated key. Zlín, Kabourek. 134-139. (in Czech and English)
6. Kippenberg H., 1981, Curculionidae: Tanymecinae. In H. Freude, K. Harde & G. A. Lohse, Die Käfer Mitteleuropas, Goecke & Evers Verlag, Krefeld, 110: 273-276.
7. Konstantinov A.S., Baselga A., Grebennikov V.V., Prena J., Lingafelter S.W., 2011, Revision of the Palearctic *Chaetocnema* species (Coleoptera: Chrysomelidae: Galerucinae: Alticini). Pensoft, Sofia. 5-363.
8. Leseigneur L., 1972, Coléoptères Elateridae de la faune de France Continentale et de Corse. Supplément au Bulletin mensuel de la Société Linnéenne de Lyon, 41 (2): 5-379.
9. Lohse G.A., 1974, Staphylinidae II (Hypocyphtinae und Aleocharinae). *In*: Freude H., Harde K., G. A. Lohse, Die Käfer Mitteleuropas, Goecke & Evers Verlag, Krefeld, 5: 1-304.
10. Lohse G.A., 1979, Dermestidae. *In:* Freude H., K. Harde, G A. Lohse, Die Käfer Mitteleuropas, Goecke & Evers, Krefeld, 6: 304-327.
11. Lohse G.A., 1983, Curculionidae: Cleoninae. *In*: Freude H., Harde K., G.A. Lohse, Die Käfer Mitteleuropas, Goecke & Evers Verlag, Krefeld, 11: 7-29.
12. Lohse G.A., 1983, Curculionidae: Barinae. *In*: Freude H., Harde K., G.A. Lohse, Die Käfer Mitteleuropas, Goecke & Evers Verlag, Krefeld, 11: 171-179.
13. LOHSE G.A., 1983, Curculionidae: Ceutorhynchinae. *In*: H. Freude, K. Harde & G. A. Lohse, Die Käfer Mitteleuropas, Goecke & Evers Verlag, Krefeld, 11: 180-253.
14. Machatschke J.W., 1969, Scarabaeidae. *In*: H. Freude, K. Harde & G. A. Lohse, Die Käfer Mitteleuropas, Goecke & Evers, Krefeld, 8: 266-366.
15. Mohr K., 1966, Chrysomelidae. *In*: H. Freude, K. W. Harde, G. A. Lohse - Die Käfer Mitteleuropas. Goeke & Evers, Krefeld, 9: 204–270.
16. Panin S., 1957, Coleoptera: Scarabaeidae. *In*: Fauna României, Editura Academiei Române, 10 (4): 1-315.
17. Panin S., Săvulescu N., 1961, Coleoptera: Cerambycidae. *In*: Fauna României. Editura Academiei Române, 10 (5): 1–523. (in Romanian)
18. Paulus H. F., 1979, Byrrhidae. *In*: Freude H., Harde K., Lohse G.A, Die Käfer Mitteleuropas, Goecke & Evers, Krefeld, 6: 328-351.
19. Reitter E., 1911, Fauna Germanica. Die Käfer des Deutschen Reiches, Band 3., Stuttgart K. G. Lutz, 1-436.
20. Reška M., 1994, Bestimmungstabellen der mitteleuropäischen Arten der Gattungen *Micrambe* Thomson und *Cryptophagus* Herbst (Insecta: Coleoptera: Cryptophagidae). Annalen des Naturhistorischen Museums in Wien, 96B: 247 – 342.
21. Telnov D., 2010, Ant-like flower beetles (Coleoptera: Anthicidae) of the UK, Ireland and Channel Isles. British Journal of Entomology and Natural History, 23: 99-117.

**Isopoda**

Radu, V.G. (1983). Ordinul Isopoda, Subordinul Oniscoidea, Oniscoidee inferioare. In Fauna R. S. R. Crustacea, **IV** (13), 1-168.

Radu, V.G. (1985). Isopoda, Oniscoidea, Crinocheta. In Fauna R. S. R., Crustacea, **IV** (14), 1-155.

**Diplopoda**

Ceuca T. (2010). Diplopoda In: In: Godeanu S. P. (red.). Determinatorul ilustrat al Florei si Faunei Romaniei, Vol. III (2) – Mediul Terestru: 290–300.

**Mites**

Mites were identified using: Balogh, 1972; Ghilyarov and Bregetova, 1977; Karg, 1993; Gerson et al. 2003; Weigmann (2006), Krantz & Walter, 2009.

1. Balogh J., 1972. The Oribatid Genera of the World. Budapest: Akademiai Kiado.
2. Gerson U., Smiley R.L., Ochoa R., 2003. Mites (Acari) for Pest Control. Blackwell Science Ltd., 558 pp. ISBN 0-632-05658-4.
3. Ghilyarov M.S., Bregetova N.G., 1977. Opredeliteľ obitayushchikh v pochve kleshcheĭ (Mesostigmata. Petrograd, Russia: Zoological Institute of the Academy of Sciences.
4. Karg W., 1993. Acari (Acarina), Milben Parasitiformes (Anactinochaeta). Cohors Gamasina Leach. Die Tierwelt Deutschlands, 59: 1-513.
5. Krantz G. W., Walter D. E., 2009. A Manual of Acarology. Third Edition. Texas Tech University Press; Lubbock, Texas, 807 pp, ISBN 978-0-89672-620-8.
6. Weigmann G. 2006. Hornmilben (Oribatida). *In*: Dahl, F. series founder. Die Tierwelt Deutschlands, part 76. Goecke & Evers, Keltern, pp. 520.

**Ants**

Ants were identified with the use of an Olympus SZ51 stereomicroscope (80 ×) and based on specific keys (Czechowski et al. 2012, Seifert 2018).

1. Czechowski, W., Radchenko, A., Czechowska, W., Vepsäläinen, K., 2012. The Ants of Poland with Reference to the Myrmecofauna of Europe. Fauna Poloniae, Vol. 4. Natura Optima Dux Foundation, Warsaw, Poland.
2. Seifert, B., 2018. The ants of Central and North Europe. Lutra Verlags- und Vertriebsgesellschaft, Tauer, Germany, pp. 408.

**Araneae**

Spiders were identified under a stereoscopic microscope with the aid of various keys (Heimer & Nentwig, 1991; Roberts, 1985; 1987; 1998, Nentwig et al., 2017). The nomenclature follows the World Spider Catalog (Platnick 2018).

1. Heimer S., Nentwig, W. 1991.SpinnenMitteleuropas. Paul PareyVerlog, Berlin und Hamburg.
2. Nentwig W, Blick T, Gloor D, Hänggi A., Kropf C. 2017. Spiders of Europe. Version 03.2017. online at: <http://www.araneae.nmbe.ch>
3. Platnick, N.I. 2018.World Spider Catalog (2018). World Spider Catalog. Version 19.5. Natural History Museum Bern, online at <http://wsc.nmbe.ch>
4. Roberts M. J. 1985. The spiders of Great Britain and Ireland, Volume 1: Atypidae to Theridiosomatidae. Harley Books, Colchester, England.
5. Roberts M. J. 1987. The Spiders of Great Britain and Ireland. Volume. 2: Linyphiidae and check list. Harley Books, Colchester, England.
6. Roberts, M. J. (1998). *Spinnengids*. Tirion, Baarn, Netherlands, 397 pp.

**Collembola**

Bretfeld G., 1999. Synopsis on Palearctic Collembola (Dunger, W., Ed.). Volume 2. Symphypleona. Abhandlungen und Berichte des Naturkundemuseums Görlitz 71(1): 1-318.

1. Dunger W., Schlitt B., 2011, Synopses on Palaearctic Collembola: Tullbergiidae. Soil Organisms 83:1-168.
2. Fjellberg A., 1998. The Collembola of Fennoscandia and Denmark. Part I: Poduromorpha. Fauna Entomol. Scand. 35, 1–183.
3. Fjellberg A., 2007. The Collembola of Fennoscandia and Denmark. Part II: Entomobryomorpha and Symphypleona. Fauna Entomol. Scand. 42, 1–264.
4. Pomorski R.J, 1998, Onychiurinae of Poland (Collembola: Onychiuridae). Genus, supplement: 201pp, Wroclaw.
5. Potapov M., 2001. Synopses on Palaearctic Collembola. Isotomidae. *In*: Abhandlungen und Berichte des Naturkundemuseums Goerlitz 73:1-603.
6. Thibaud J.M., Schulz H.J., da Gama Assalino M.M., 2004, Synopses on Palaearctic Collembola. Hypogastruridae. *In*: Abhandlungen und Berichte des Naturkundemuseums. Goerlitz 75: 1-287.
