# Supplementary material for: Effects of vineyard inter-row management on the diversity and abundance of plants and surface-dwelling invertebrates in Central Romania
Source: J Insect Conserv. 2020 Jan 14;24(1):175–85. doi: 10.1007/s10841-019-00215-0 (PMC7002328; doi:10.1007/s10841-019-00215-0)
Supplement: Supplementary file 4 — Supplementary file4 (DOCX 25 kb) [file 10841_2019_215_MOESM4_ESM.docx]

**Effects of vineyard inter-row management on the diversity and abundance of plants and surface-dwelling invertebrates in Central Romania**

Cristina Fiera^1^, Werner Ulrich^2^, Daniela Popescu^3,4^, Claudiu-Ioan Bunea^4^, Minodora Manu^1^, Ioana Nae^5^, Melania Stan^6^, Bálint Markó^7^, István Urák^8^, Andrei Giurginca^5^, Nicole Penke^9^, Silvia Winter^9,10^, Sophie Kratschmer^9,11^, Jacob Buchholz^11^, Pascal Querner^11^, Johann G. Zaller^11^*

***S4. References used in TRY database***

1. Bragazza, L., 2009. Conservation priortiy of Italian alpine habitats: a floristic approach based on potential distribution of vascular plant species. Biodiversity and Conservation, 18: 2823–2835.
2. Burrascano, S., Copiz, R., Del Vico, E., Fagiani, S., Giarrizzo, E., Mei, M., Mortelliti, A., Sabatini, F. M., Blasi, C., 2015. Wild boar rooting intensity determines shifts in understorey composition and functional traits, Community Ecology 16(2): 244-253.
3. Campbell, C., Atkinson, L., Zaragoza-Castells, J., Lundmark, M., Atkin, O., Hurry, V., 2007. Acclimation of photosynthesis and respiration is asynchronous in response to changes in temperature regardless of plant functional group. New Phytologist 176: 375-389.
4. Campetella, G., Botta-Dukát, Z., Wellstein, C., Canullo, R., Gatto, S., Chelli, S., Mucina, L., Bartha, S., 2011. Patterns of plant trait-environment relationships along a forest succession chronosequence. Agriculture, Ecosystems & Environment, 145(1): 38-48, doi:10.1016/j.agee.2011.06.025.
5. Ciccarelli D., 2015. Mediterranean coastal dune vegetation: Are disturbance and stress the key selective forces that drive the psammophilous succession? Estuarine, Coastal and Shelf Science 165(5):247-253 doi: 10.1016/j.ecss.2015.05.023
6. Ciocarlan V., 2009. The illustrated Flora of Romania, Pteridophyta et Spermatopyta, Editura Ceres, 1141 pp.
7. Cornelissen, J. H. C., 1996. An experimental comparison of leaf decomposition rates in a wide range of temperate plant species and types, Journal of Ecology 84: 573-582.
8. Cornelissen, J. H. C., Cerabolini, B., Castro-Diez, P., Villar-Salvador, P., Montserrat-Marti, G., Puyravaud, J. P., Maestro, M., Werger, M. J. A., Aerts, R., 2003. Functional traits of woody plants: correspondence of species rankings between field adults and laboratory-grown seedlings? Journal of Vegetation Science 14: 311-322.
9. De Vries F., Bardgett R.D., 2016. Plant community controls on short-term ecosystem nitrogen retention. New Phytologist. doi: 10.1111/nph.13832.
10. Díaz, S., Hodgson, J. G., Thompson, K., Cabido, M., Cornelissen, J. H. C., Jalili, A., Montserrat-Martí, G., Grime, J. P., Zarrinkamar, F., Asri, Y., Band, S. R., Basoncelo, S., Castro-Díez, P., Funes, G., Hamzehee, B., Khoshnevi, M., Pérez-Harguindeguy, N., Pérez-Rontomé, M. C., Shirvany, F. A., Vendramini, F., Yazdani, S., Abbas-Azimi, R., Bogaard, A., Boustani, S., Charles, M., Dehghan, M., de Torres-Espuny, L., Falczuk, V., Guerrero-Campo, J., Hynd, A., Jones, G., Kowsary, E., Kazemi-Saeed, F., Maestro-Martínez, M., Romo-Díez, A., Shaw, S., Siavash, B., Villar-Salvador, P., and Zak, M. R. (2004): The plant traits that drive ecosystems: Evidence from three contintents. Journal of Vegetation Science 15: 195-304.
11. Everwand G., Fry, E. L., Eggers, T., Manning, P., 2014. Seasonal variation in the relationship between plant traits and grassland carbon and water fluxes. Ecosystems 17, 1095-1108.
12. Fitter, A. H., Peat, H. J., 1994. The Ecological Flora Database, Journal of Ecology 82: 415-425.
13. Fonseca, C. R., Overton, J. M., Collins, B., Westoby, M., 2000. Shifts in trait-combinations along rainfall and phosphorus gradients. Journal of Ecology, 88: 964-977.
14. Frenette-Dussault, C., Shipley, B., Lúger, J. F., Meziane, D., Hingrat, Y., 2012. Functional structure of an arid steppe plant community reveals similarities with Grime's C-S-R theory. Journal of Vegetation Science 23:208-222.
15. Fry, E. L., Power, S. A., Manning, P., 2014. Trait based classification and manipulation of functional groups in biodiversity-ecosystem function experiments, Journal of Vegetation Science, 25: 248-261.
16. Gachet, S., Véla, E., Tatoni, T., 2005. BASECO: a floristic and ecological database of Mediterranean French flora, Biodiversity and Conservation 14(4): 1023-1034.
17. Giarrizzo, E., Burrascano, S., Chiti, T., de Bello, F., Lepš, J., Zavattero, L., and Blasi, C., 2016. Re-visiting historical semi-natural grasslands in the Apennines to assess patterns of changes in species composition and functional traits. Applied Vegetation Science, doi:10.1111/avsc.12288.
18. Gos P., Loucougaray G., Colace, M. P., Arnoldi, C., Gaucherand, S., Dumazel, D., Girard, L., Delorme, S., Lavorel, S., 2016. Oecologia 180: 1001 doi:10.1007/s00442-016-3551-3
19. Green, W., 2009. USDA PLANTS Compilation, version 1, http://bricol.net/downloads/data/PLANTSdatabase/) NRCS: The PLANTS Database (http://plants.usda.gov. 1 Feb 2009), National Plant Data Center: Baton Rouge, LA 70874-74490, USA.
20. Hattermann, D., Elstner, C., Bernhardt-Römermann, M., Eckstein, L. Measurements from the project “Relative effects of local and regional factors as drivers for plant community diversity, functional trait diversity and genetic structure of species on Baltic uplift islands” funded by the German Research Foundation - DFG: BE 4143/5-1 and EC 209/12-1.
21. Hill, M. O., Preston, C. D., Roy, D. B., 2004. PLANTATT - attributes of British and Irish Plants: status, size, life history, geography and habitats, Huntingdon: Centre for Ecology and Hydrology
22. Kazakou, E., Vile, D., Shipley, B., Gallet, C., Garnier, E., 2006. Co-variations in litter decomposition, leaf traits and plant growth in species from a Mediterranean old-field succession. Functional Ecology 20: 21-30.
23. Kichenin, E., Wardle, D. A., Peltzer, D. A., Morse, C. W., Freschet, G. T., 2013. Contrasting effects of plant inter- and intraspecific variation on community-level trait measures along an environmental gradient. Functional Ecology, 27(5), doi:10.1111/1365-2435.12116.
24. Kleyer M., Bekker, R. M., Knevel, I. C., Bakker, J. P., Thompson, K., Sonnenschein, M., Poschlod P., Van Groenendael, J. M., Klimes, L., Klimesová, J., Klotz, S., Rusch, G. M., Hermy, M., Adriaens, D., Boedltje, G., Bossuyt, B., Dannemann, A., Endels, P., Götzenbeger, L., Hodgson, J. G., Jackel, A.-K., Kühn, I., Kunzmann, D., Ozinga, W. A., Römermann, C., Stadler, M., Schlegelmilch, J., Steendam, H. J., Tackenberg, O., Wilmann, B., Cornelissen, J. H. C., Eriksson, O., Garnier, E., Peco, B., 2008.
    The LEDA Traitbase: A database of life-history traits of Northwest European flora. Journal of Ecology 96: 1266-1274.
25. La Pierre, K. J., Smith, M. D., 2015. Functional trait expression of grassland species shift with short- and long-term nutrient additions. Plant Ecology 216: 307, doi:10.1007/s11258-014-0438-4
26. Laughlin, D. C., Leppert, J. J., Moore, M. M., Sieg, C. H., 2010. A multi-trait test of the leaf-height-seed plant strategy scheme with 133 species from a pine forest flora. Functional Ecology 24: 493-501
27. Lhotsky B., Csecserits, A., Kovacs, B., Botta-Dukát, Z.: New plant trait records of the Hungarian flora. Acta Botanica Hungarica 59: 397-400.
28. Louault, F., Pillar, V.D., Aufrère, J., Garnier, E., Soussana, J.F., 2005. Plant traits and functional types in response to reduced disturbance in a semi-natural grassland. Journal of Vegetation Science 16, 151-160.
29. Loveys, B. R., Atkinson, L. J., Sherlock, D. J., Roberts, R. L., Fitter, A. H., Atkin, O. K., 2003. Thermal acclimation of leaf and root respiration: an investigation comparing inherently fast- and slow-growing plant species. Global Change Biology 9: 895-910.
30. Meziane, D., Shipley, B., 1999. Interacting determinants of specific leaf area in 22 herbaceous species: effects of irradiance and nutrient availability. Plant Cell and Environment 22: 447-459.
31. Minden V., Andratschke, S., Spalke, J., Timmermann, H., Kleyer, M. 2012. Plant trait-environment relationships in salt marshes: Deviations from predictions by ecological concepts. Perspectives in Plant Ecology, Evolution and Systematics 14: 183-192.
32. Minden, V., Kleyer, M., 2015. Ecosystem multifunctionality of coastal marshes is determined by key plant traits. Journal of Vegetation Science 26: 651-662.
33. Ordonez, J. C., van Bodegom, P. M., Witte, J. P. M., Bartholomeus, R. P., van Hal, J. R., Aerts, R., 2010. Plant Strategies in Relation to Resource Supply in Mesic to Wet Environments: Does Theory Mirror Nature? American Naturalist 175 :225-239.
34. Paula, S., Arianoutsou, M., Kazanis, D., Tavsanoglu, Ç., Lloret, F., Buhk, C., Ojeda, F., Luna, B., Moreno, J. M., Rodrigo, A., Espelta, J. M., Palacio, S., Fernández-Santos, B., Fernandes, P. M., Pausas, J. G., 2009. Fire-related traits for plant species of the Mediterranean Basin, Ecology 90:1 420.
35. Peco B., de Pablos, I., Traba, J., Levassor, C., 2005. The effect of grazing abandonment on species composition and functional traits: the case of dehesa Basic and Applied Ecology 6(2): 175-183.
36. Pierce S., Vagge, I., Brusa, G., Cerabolini, B. E. L., 2014. The intimacy between sexual traits and Grime’s CSR strategies for orchids coexisting in semi-natural calcareous grassland at the Olive Lawn. Plant Ecology 215(5): 495-505.
37. Prentice, I. C., Meng, T., Wang, H., Harrison, S. P., Ni, J., Wang, G., 2011. Evidence for a universal scaling relationship of leaf CO_2_ drawdown along a moisture gradient. New Phytologist 190: 169–180.
38. Sandel, B., Corbin, J. D., Krupa, M., 2011. Using plant functional traits to guide restoration: A case study in California coastal grassland. Ecosphere 2(2), doi:10.1890/ES10-00175.1.
39. Schroeder-Georgi, T., Wirth, C., Nadrowski, K., Meyer, S. T., Mommer, L., Weigelt, A., 2016. From pots to plots: hierarchical trait-based prediction of plant performance in a mesic grassland, Journal of Ecology 104: 206–218, doi:10.1111/1365-2745.12489.
40. Sheremetev, S. N., 2005. Herbs on the soil moisture gradient (water relations and the structural-functional organization). KMK, Moscow, 271 pp.
41. Shipley, B., Vu, T. T., 2002. Dry matter content as a measure of dry matter concentration in plants and their parts. New Phytologist 153: 359-364.
42. Siefert, A., Fridley, J. D., Ritchie, M. E., 2014. Community functional responses to soil and climate at multiple spatial scales: when does intraspecific variation matter? PLOS ONE 9: e111189.
43. Smith, S. W., Woodin, S. J., Pakeman, R. J., Johnson, D., van der Wal, R., 2014. Root traits predict decomposition across a landscape-scale grazing experiment. New Phytologist. doi: 10.1111/nph.12845.
44. Takkis, K., 2014. Changes in plant species richness and population performance in response to habitat loss and fragmentation. Dissertation, University of Tartu. TARTUENSIS 255, 2014-04-07; Available from: <http://hdl.handle.net/10062/39546>.
45. Tribouillois, H., Fort, F., Cruz, P., Charles, R., Flores, O., Garnier, E., 2015. A Functional Characterisation of a Wide Range of Cover Crop Species: Growth and Nitrogen Acquisition Rates, Leaf Traits and Ecological Strategies. PLoS ONE 10(3): e0122156, doi:10.1371/journal.pone.0122156.
46. Wirth, C., Lichstein, J. W., 2009. The Imprint of Species Turnover on Old-Growth Forest Carbon Balances - Insights From a Trait-Based Model of Forest Dynamics, Pages 81-113 in Wirth, C., Gleixner, G., Heimann, M. (editors), Old-Growth Forests: Function, Fate and Value, Springer, New York, Berlin, Heidelberg.
47. Wright, I. J., Reich, P. B., Westoby, M., Ackerly, D. D., Baruch, Z., Bongers, F., Cavender-Bares, J., Chapin, T., Cornelissen, J. H. C., Diemer, M., Flexas, J., Garnier, E., Groom, P. K., Gulias, J., Hikosaka, K., Lamont, B. B., Lee, T., Lee, W., Lusk, C., Midgley, J. J., Navas, M. L., Niinemets, U., Oleksyn, J., Osada, N., Poorter, H., Poot, P., Prior, L., Pyankov, V. I., Roumet, C., Thomas, S. C., Tjoelker, M. G., Veneklaas, E. J., Villar, R., 2004. The worldwide leaf economics spectrum. Nature 428: 821-827.
